# The Impact of Hypoglycemia on Patients with Diabetes Mellitus: A Cross-Sectional Analysis

**DOI:** 10.3390/jcm11030626

**Published:** 2022-01-26

**Authors:** Siddarth Agrawal, Sebastian Makuch, Mateusz Dróżdż, Tomasz Dudzik, Igor Domański, Rafał Poręba, Grzegorz Mazur

**Affiliations:** 1 Department and Clinic of Internal Medicine, Occupational Diseases, Hypertension and Clinical Oncology, Wroclaw Medical University, 50-556 Wroclaw, Poland; rafal.poreba@umed.wroc.pl (R.P.); grzegorz.mazur@umed.wroc.pl (G.M.); 2 Department of Clinical and Experimental Pathology, Wroclaw Medical University, 50-368 Wroclaw, Poland; sebastian.mk21@gmail.com; 3 Faculty of Medicine, Wroclaw Medical University, Jana Mikulicza-Radeckiego 5, 50-345 Wroclaw, Poland; mateuszdrozdz5208@gmail.com (M.D.); tomasz.dudzik@student.umed.wroc.pl (T.D.); igor.domanski@student.umed.wroc.pl (I.D.)

**Keywords:** hypoglycemia, diabetes mellitus, risk factors

## Abstract

The increasing mortality and morbidity in patients with diabetes mellitus constitute a severe public health problem. The condition is recognized as a cause of impaired quality of life, high costs, and diminished productivity. In this study, we performed a cross-sectional analysis among 300 Polish participants with type 1 and type 2 diabetes to determine and classify risk factors associated with increased incidences of hypoglycemia. Including an open-access knowledge about the correlations between diabetes rates and human’s lifestyle, we confirm that the frequency of smoking and drinking alcohol, low BMI, inappropriate diet, low physical activity, lack of vaccination against influenza and pneumococci, and co-existence of other comorbidities such as cardiovascular diseases, thyroid diseases, hyperlipidemia, retinopathy, and asthma elevate the risk of hypoglycemia. Furthermore, hypoglycemic patients were more often malnourished, depressed, irritated, and exposed to stress. In sum, the analysis of the interaction between diabetes and sociodemographic, environmental, or other disease-related risk factors provides strategies to optimize glycemic control and reduce the incidence of hypoglycemia. Furthermore, we believe our findings may constitute a basis for promoting health by adjusting available and implementing new preventive services reducing hypoglycemic episodes in diabetic patients.

## 1. Introduction

Year by year, the prevalence of diabetes mellitus (DM) is continuously growing [1]. This disease can be classified into two categories: type 1 diabetes, in which insulin is no longer produced by pancreatic β cells because of their malfunction, and type 2 diabetes, characterized by the increase of insulin resistance, leading to hyperglycemia [2]. Type 2 diabetes is associated with more severe complications, including cardiovascular and renal failure, retinopathy with increased risk of blindness, diabetic foot potentially leading to amputations, and severe infections [3]. Furthermore, several studies indicated that DM harms the quality of life. The prevalence rates of depression are up to three times higher in patients with type 1 diabetes and twice as high in people with type 2 diabetes than the general population worldwide [4,5,6,7,8]. Although numerous articles focus on the epidemiology, complications, therapies, and health strategies of diabetes mellitus, literature data regarding the proper management of blood glucose levels are still scarce.

Iatrogenic complications of inappropriate insulin dose administrations can lead to hypoglycemia [9]. According to the American Diabetes Association, this condition is abnormally low plasma glucose concentration that exposes the individual to potential harm [10]. It is estimated that patients with type 1 diabetes suffer from symptomatic hypoglycemic episodes twice a week and at least once a year with a serious event. Approximately 2–4% of deaths of DM patients are due to hypoglycemic episodes. However, type 2 DM patients have substantially fewer hypoglycemic episodes than those with type 1 diabetes [11].

Numerous studies have shown that inaccurate management of blood glucose levels may increase the risk of cardiovascular symptoms [12,13,14]. In more severe stages, due to the deprivation of the central nervous system, individuals with hypoglycemia require the assistance of another person who actively administers medications and/or carbohydrates to increase blood glucose levels [15]. Nevertheless, it is worth noting that symptoms of hypoglycemia and their severity may vary between patients or may occur differently every time [16].

Two different pathophysiological processes trigger hypoglycemia. The first process is characterized by decreased glucose levels in the blood. This condition is referred to as a hypoglycemic episode induced by catecholamines’ release, leading to neurogenic symptoms [11,12,13,14,15,16,17,18]. Some include shakiness, anxiety, nervousness, sweating, or pallor [11,19,20]. Moreover, acetylcholine is released from postsynaptic sympathetic nerve endings and may develop additional symptoms, including diaphoresis, hunger, and parentheses [11,19,21]. The second pathophysiological process is neuroglycopenia. This condition results from the brain neuronal glucose deprivation [11,19,20,21] and is mainly characterized by ataxia, confusion, speaking problems, seizures, coma, and in most severe complications, death [11,21]. In this study, we made an effort to find and classify factors that increase the risk of hypoglycemic episodes in patients suffering from diabetes mellitus. The long-term perspective of this study is to provide more personalized management of individuals with a high incidence of hypoglycemia and improve their chronic care.

## 2. Materials and Methods

### 2.1. Study Design and Participants

The cross-sectional analysis included 300 participants (144 women and 156 men) with type 1 and type 2 diabetes and was performed between July and October 2021. Out of 300 participants, 184 were treated with metformin or other antidiabetic drugs (patients with type 2 diabetes), and 116 were treated with insulin (patients with type 1 diabetes). All patients aged 18 years old and above were interviewed during medical consultations based on the structured questionnaire adapted from Stanford Patients Education Research Center [22], translated, and pre-tested for use in the Polish population. The questionnaire contains questions about demographic data, disease history, general health status in terms of the occurrence of hypoglycemic symptoms, utilization of clinical preventive services, willingness to use available online medical consultations, and health-related behaviors. The studied group included outpatients and inpatients from different locations in Poland. The inclusion criteria were as follows: (1) aged 18 years or older; (2) a diabetes diagnosis with no other serious complications; (3) able to read and write in Polish. The non-hypoglycemia group included individuals who answered “0 times” to the question “how many times in the last year have you experienced severe events related to low blood sugar, including loss of consciousness or need for help”? and “0 times or 1–3 times” to the question: “How many times in the last month have you experienced symptoms due to low blood sugar, including sweating, weakness, anxiety, tremor, hunger, or headache” (Table S1).

### 2.2. Variables

The variables of interest in this study are age, gender, urban/rural residence, the knowledge about diabetes, type of diabetes, participants’ body mass index (BMI kg/m^2^) calculated as mass (kg) divided by the square of height (m^2^), willingness to use mobile health monitoring applications, the level of trust in medical context and interpretation of laboratory tests, the presence of chronic non-communicable diseases such as hyperlipidemia, diabetic retinopathy, and asthma, use of medication for controlling diabetes, current smoking and alcohol consumption status, vaccination status, foot self-care behavior, use of special diet, and physical activity. No compensation was provided for participating in this study. This study was approved by the Bioethics Committee of Wroclaw Medical University.

### 2.3. Statistics

Data were evaluated using Statistica v.13.3 (TIBCO Software Inc. Palo Alto, Santa Clara, CA, USA). Descriptive statistics were calculated for continuous quantitative variables, and the non-parametric significance test (Mann-Whitney U) was applied for qualitative variables (nominal and ordinal), the numbers (*n*) and structure indexes (%) were calculated, and chi-square tests of independence were used. Univariate and multivariate logistic regression models were used to evaluate the relationships between the risk of hypoglycemia and anthropogenic, and clinical factors and disease-related behaviors among the studied population, with adjustment for potential confounding factors (including age, sex, housing condition, net income, BMI, chronic diseases, time spent in computer/smartphones/tablets, the knowledge about diabetes, the level of trust in medical content, and interpretation of laboratory tests), yielding adjusted odds ratios (ORs) and 95% CIs. A *p*-value of less than 0.05 was considered statistically significant for the statistical hypothesis testing.

## 3. Results

Hypoglycemia was reported in 186 cases of the study group (186/300; 62.0%), more frequent in men than in women (102/186; 54.8%) and in individuals aged <55 years old than at later ages (111/186; 59.7%) (Table S1). Respondents were also asked to self-assess their knowledge about diabetes, satisfaction with the medical care they receive for the illness or not, and the willingness to use internet-connected applications providing medical consultations (Table S2). 163 individuals had severe low blood sugar events, including passing out or needing help at least once in the last year (163/186; 87.6%, *p* < 0.001, Table S2), and 69 patients had high blood sugar with symptoms such as thirst, dry mouth, and skin, increased urine sugar, decreased appetite, nausea or tiredness for continuously four days or more in the last month (69/186; 37.1% *p* < 0.001, Table S2). Considering patients with a different type of diabetes, those with type 2 diabetes are more likely to have hypoglycemia if they have symptoms such as thirst, dry mouth, increased urine sugar, decreased appetite, nausea, or tiredness that occur continuously at least four days last month (*p* < 0.001; OR = 5.28, CI95% [2.08–13.4], Table S2b vs. 0.002; OR = 4.33, CI95% [1.62–11.5], Table S2a).

**Figure 1:**
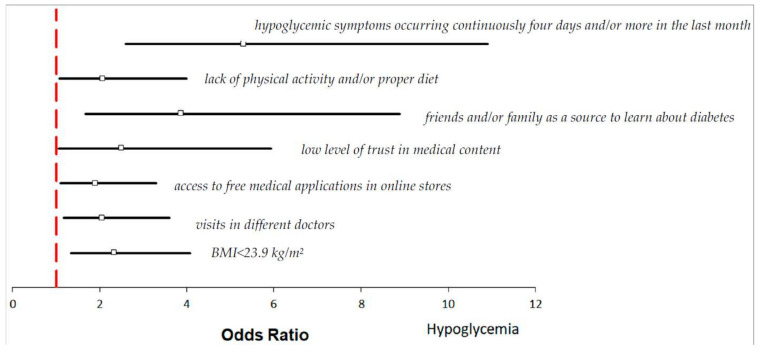
Odds ratios and 95% confidence interval for the relationships between the risk of hypoglycemia and anthropogenic, clinical, and physical factors across the entire group (more details in Tables S1–S3).

Figure 1 shows the results of multivariate logistic regression analysis, with adjustment for all potential confounding factors in the 186 hypoglycemic participants. The risk of hypoglycemia is significantly associated with low BMI < 23.9 kg/m^2^ (*p* = 0.003; OR = 2.34, CI95% [1.34–4.08], Table S3). Furthermore, respondents reporting different doctors’ visits (*p* = 0.011; OR = 2.06, CI95% [1.18–3.60], Table S3) have hypoglycemia nearly twice more often than those visiting permanently the same GP doctor. If mobile medical applications are free and accessible in online stores, they would be used by 99 respondents (99/186; 53.2%, *p* = 0.004, Table S2), and this action may increase the risk of hypoglycemia approximately twofold (*p* = 0.022; OR = 1.90, CI95% [1.10–3.30], Table S3) and threefold in the case of patients with type 2 diabetes (*p* = 0.011; OR = 3.25, CI95% [1.31–8.06], Table S3b). The other behavior enhancing hypoglycemic episodes is lack of trust in medical content, interpretation of laboratory tests, and description of ailments available on the internet (*p* = 0.037; OR = 2.51, CI95% [1.06–5.94], Table S3). Moreover, the risk of hypoglycemia increases in those who believe the best source to learn about diabetes is from friends and/or family members (*p* = 0.001; OR = 3.87; CI95% [1.68–8.88], Table S3), in those who do not follow any recommendations regarding non-pharmacological treatment, in particular regarding physical activity and a proper diet (*p* = 0.03; OR = 2.07, CI95% [1.07–4.00], Table S3), and in those who in the last month had high blood sugar levels with hypoglycemic symptoms including thirst, dry mouth, and skin, increased urine sugar, decreased appetite, nausea or tiredness for at least four days (*p* < 0.001; OR = 5.31, CI95% [2.60–10.9], Table S3). If patients with type 2 diabetes have these symptoms, there is an approximately 8 times higher risk of suffering from hypoglycemia (*p* = 0.008; OR = 8.52, CI95% [2.30–31.5], Table S3b). Thus, the continuous occurrence of hypoglycemic symptoms has a crucial impact on the frequency of this disease; in this case, the logistic regression coefficient “b” was the highest (b = 1.67, Table S3).

**Figure 2:**
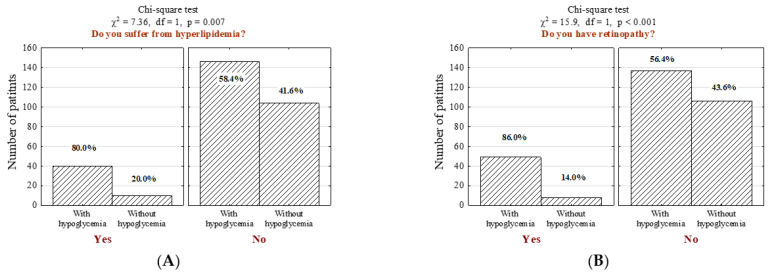

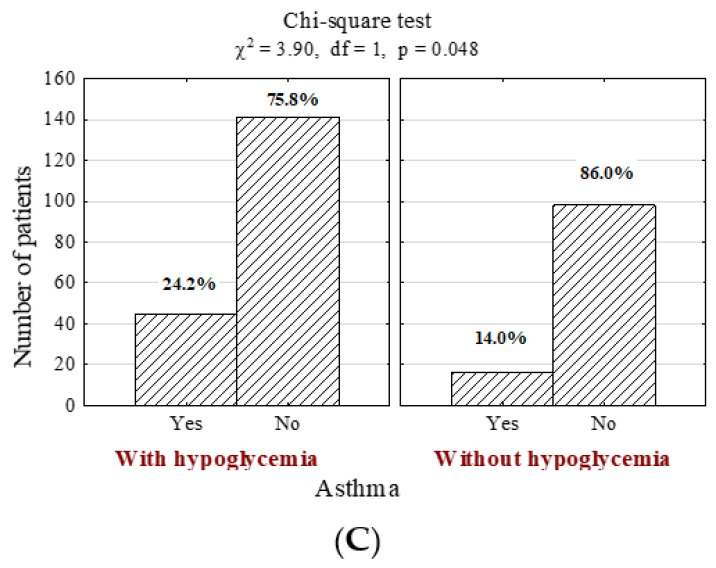
Number (*n*) and percentage (%) of patients in groups differing in the presence of hypoglycemia and (**A**) hyperlipidemia, (**B**) diabetic retinopathy, (**C**) asthma, and the results of the independence tests.

Furthermore, individuals with hypoglycemia were more likely to suffer from other comorbidities, including hyperlipidemia (Figure 2A; *n* = 40; 21.5% vs. 8.8%, *p* = 0.007), diabetic retinopathy (Figure 2B; *n* = 57; 26.3% vs. 7.0%, *p* <0.001) and asthma (Figure 2C; *n* = 61; 24.2% vs. 14.0%, *p* = 0.048), even after the adjustment of potential confounders (*p* = 0.048; OR = 1.95, CI95% [1.05–3.66], Table S1).

**Figure 3:**
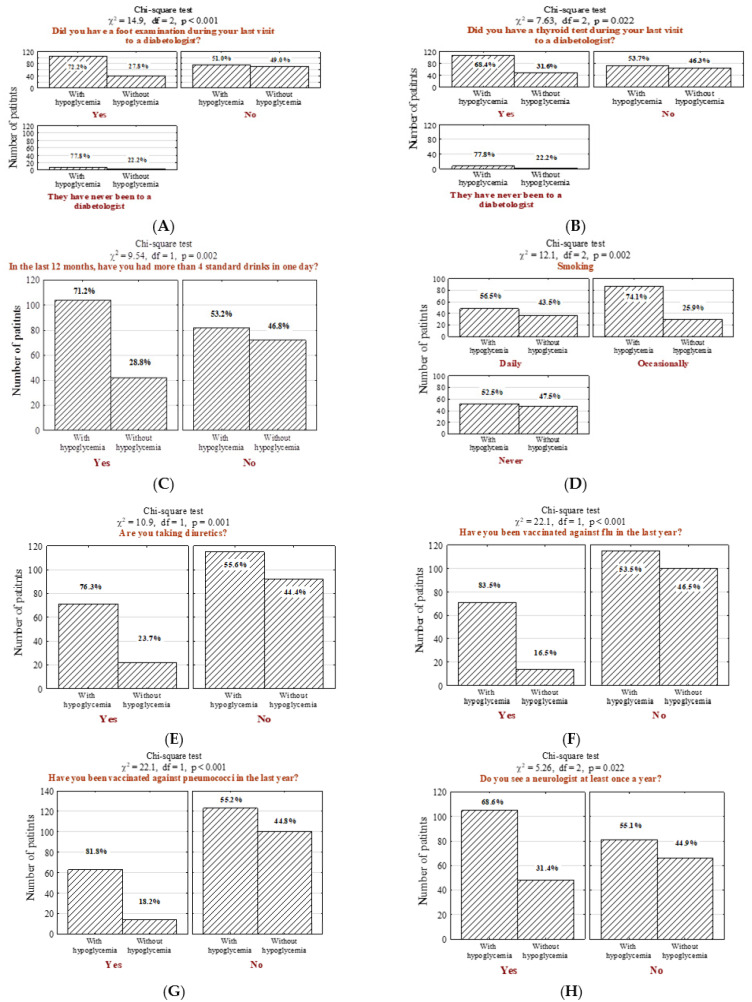
Number (*n*) and percentage (%) of patients in groups differing in the presence of hypoglycemia and foot examination by the diabetic at the last visit (**A**), thyroid examination by the diabetic at the last visit (**B**), drinking more than 4 standard drinks per day in the last 12 months (1 standard drink is referred to as a glass of 250 mL of 5% alcoholic beer; a glass of 100 mL of 12% alcoholic wine; a bottle of 30 mL of 40% alcoholic vodka) (**C**), smoking cigarette (**D**), the intake of diuretics (**E**), influenza vaccination in the last year (**F**), pneumococcal vaccination (**G**), annual follow-up visit with a neurologist (**H**), and the results of the independence tests. Results were generated before the adjustment of potential confounders.

Patients with high incidence of hypoglycemia were more likely to undergo diabetic foot examination (Figure 3A; *n* = 104; 55.9% vs. 35.1%, *p* < 0.001) as well as thyroid examination (Figure 3B; *n* = 106; 57% vs. 43%, *p* = 0.022). Hypoglycemic patients were more often identified as addicted to alcohol (more than four alcoholic drink-equivalent drinks per day in the last period of 12 months) (Figure 3C; *n* = 104; 55.9% vs. 36.8%, *p* = 0.002) and nicotine (Figure 3D; *n* = 52; 28% vs. 41.2, *p* = 0.002). Moreover, individuals suffering from hypoglycemia were more often on antihypertensive therapy (Figure 3E; *n* = 71; 38.2% vs. 19.3%, *p* < 0.001) and vaccinated against influenza (Figure 3F; *n* = 71; 38.2% vs. 12.3%, *p* < 0.001) and pneumococci (Figure 3G; *n* = 63; 33.9% vs. 12.3%, *p* < 0.001). In addition, they underwent neurologist consultations more frequently (Figure 3H; *n* = 105; 56.5% vs. 42.1%, *p* = 0.022).

**Figure 4:**
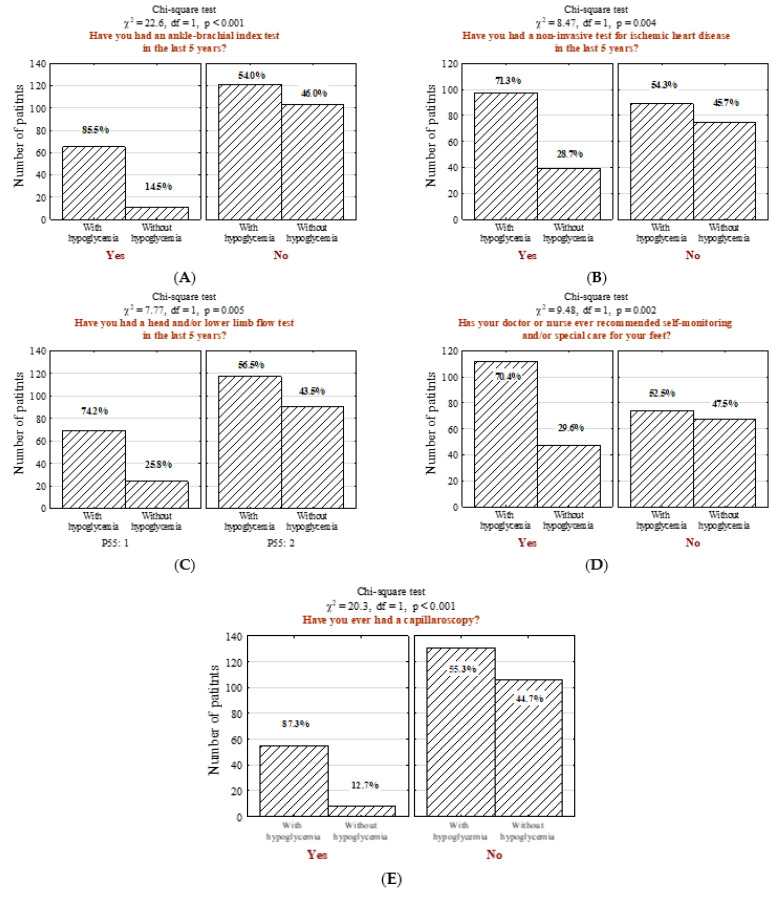
Number (*n*) and percentage (%) of patients in groups differing in the presence hypoglycemia and (**A**) ankle-brachial index test, (**B**) non-invasive ischemic heart disease tests, (**C**) examination to assess the intracerebral and/or lower limb flows, (**D**) self-monitoring and/or special care recommendation for their feet, (**E**) capillaroscopy and the results of the independence tests.

In the studied questionnaire, respondents were also asked if their attending physician performed several specific medical examinations in the last five years. It was determined that people with hypoglycemia more often underwent ankle-brachial index measurement (Figure 4A; *n* = 65; 34.9% vs. 9.6%, *p* < 0.001), non-invasive examination for ischemic heart disease (Figure 4B; *n* = 97; 52.2% vs. 34.2%, *p* = 0.004), and doppler ultrasound test of carotid or femoral blood flow (Figure 4C; *n* = 69; 37.1% vs. 21.1%, *p* = 0.005). Moreover, a significant majority of surveyed patients with hypoglycemia performed self-control and/or special foot self-care (Figure 4D; *n* = 112; 60.2% vs. 41.2%, *p* = 0.002) and underwent non-invasive capillaroscopy (Figure 4E; *n* = 55; 29.6% vs. 7.0%, *p* < 0.001).

**Figure 5:**
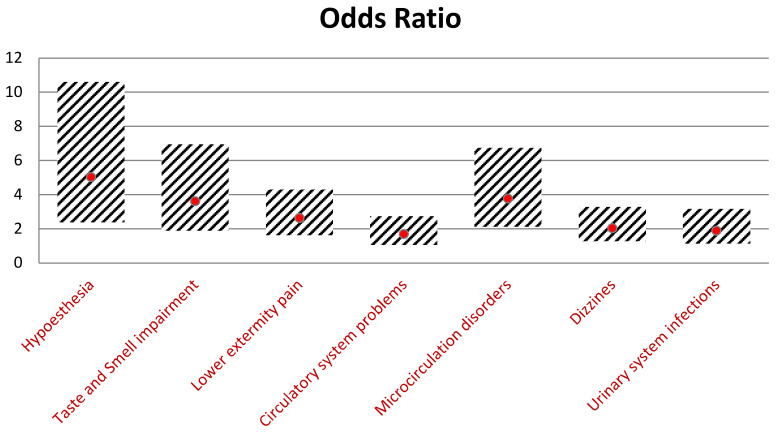
Comparison of the odds ratios of symptoms and their 95% confidence intervals.

Including 186 individuals with hypoglycemia, we determined which symptoms are the most crucial in making a proper diagnosis. Patients with hypoglycemia were five times more likely to suffer from hypoesthesia (*p* < 0.001; OR = 5.03), 3.61 times more likely to report smell and taste impairment (*p* < 0.001; OR = 3.61), and 2.6 times more likely to feel pain in the lower limb (*p* < 0.001; OR = 2.64). Furthermore, hypoglycemia symptoms occurred more frequently among patients with cardiovascular and microcirculation disorders (*p* = 0.041; OR = 1.7 and *p* < 0.001; OR = 3.77, respectively). Individuals with a high incidence of hypoglycemia reported dizziness almost two times higher than non-hypoglycemic individuals. The statistical significance was also observed in hypoglycemic individuals with frequently recurrent urinary tract infections (UTI) (*p* = 0.02 OR = 1.89). Detailed data analyzing patients with a greater tendency to suffer from hypoglycemia are presented in Figure 5.

**Figure 6:**
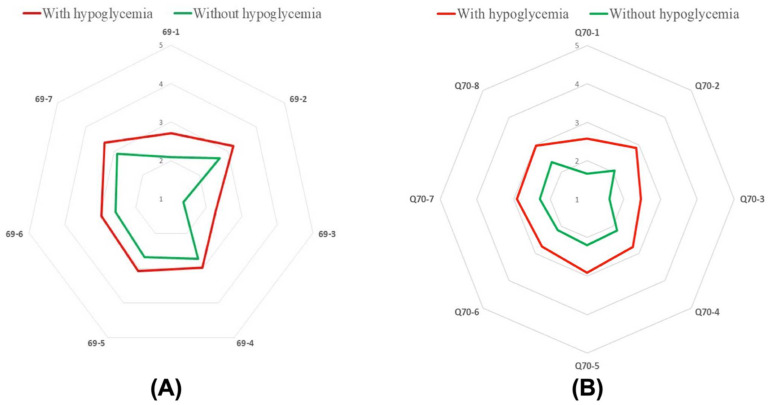
A median number of patients in group differing in the presence of hypoglycemia and too high blood sugar level caused by: infection (**A**; 69-1), feeling irritated (**A**; 69-2), taking the wrong dose of medication (**A**; 69-3), eating the wrong type of food (**A**; 69-4), overeating (**A**; 69-5), lower physical activity (**A**; 69-6), stress (**A**; 69-7). A median number of patients in group differing in the presence of hypoglycemia and too low blood sugar level caused by: infection (**B**; 70-1), feeling irritated (**B**; 70-2), taking the wrong dose of medication (**B**; 70-3), eating the wrong type of food (**B**; 70-4), overeating (**B**; 70-5), lower physical activity (**B**; 70-6), skipping a meal (**B**; 70-7), stress (**B**; 70-8).

Several external factors influence the proper regulation of blood sugar levels, including physical activity, diet, and stress. It is predominantly important for patients with diabetes who are recommended to adjust their lifestyle for effective treatment. In our study, we identified groups of patients for whom the risk of the maintenance of proper blood glucose levels is the most challenging. At first, we analyzed hypoglycemic patients with too-high blood sugar levels. We showed that the incidence of infection (Figure 6A, 69-1) (M ± SD 2.7 ± 0.9 to M ± SD 2.1 ± 1.0, *p* < 0.001), feeling irritated (Figure 6A, 69-2) (M ± SD 3.2 ± 1.2 to M ± SD 2.7 ± 1.1, *p* < 0.001), taking a too low amount of injected insulin or oral hypoglycemic drugs (Figure 6A, 69-3) (M ± SD 2.3 ± 1.3 to M ± SD 1.3 ± 0.7, *p* < 0.001), inappropiate high-calorific diet (Figure 6A, 69-4) (M ± SD 3.0 ± 1.1 to M ± SD 2.7 ± 1.1 *p* = 0.02), overeating (Figure 6A, 69-5) (M ± SD 3.1 ± 1.1 to M ± SD 2.7 ± 1.1, *p* = 0.001), exhibiting less physical activity than usual (Figure 6A, 69-6) (M ± SD 3.0 ± 1.1 to M ± SD 2.1 6 ± 1.0 *p* = 0.002), and stress exposure (Figure 6A, 69-7) (M ± SD 3.3 ± 1.2 to M ± SD 2.9 ± 1.2, *p* = 0.002) are factors increasing the risk of too high-blood sugar levels in hypoglycemic patients compared to the non-hypoglycemic group.

Then we analyzed hypoglycemic patients with too-low blood glucose levels. In this case, we also found that the incidence of infection (Figure 6B, 70-1) (M ± SD 2.6 ± 1.1, to M ± SD 1.7 ± 0.9, *p* < 0.001), feeling irritated (Figure 6B, 70-2) (M ± SD 2.9 ± 1.2 to M ± SD 2.1 ± 1.1, *p* < 0.001), taking the wrong dose of medicine (in this case too high amount of injected insulin or oral hypoglycemic drugs) (Figure 6B, 70-1) (M ± SD 2.5 ± 1.3 to M ± SD 1.6 ± 0.9 *p* < 0.001), inappropiate low-calorific diet (Figure 6B, 70-4) (M ± SD 2.8 ± 1.1 to M ± SD 2.1 ± 1.1, *p* < 0.001), overeating (Figure 6B, 70-5) (M ± SD 2.9 ± 1.1 to M ± SD 2.2 ± 1.0, *p* < 0.001), less physical activity than usual (Figure 6B, 70-6) (M ± SD 2.7 ± 1.1 to M ± SD 2.1 ± 1.2, *p* < 0.001), skipping a meal (Figure 6B, 70-7)(M ± SD 2.9 ± 1.1 to M ± SD 2.3 ± 1.1, *p* < 0.001) and stress exposure (Figure 6B, 70-8) (M ± SD 3.0 ± 1.2 to M ± SD 2.4 ± 1.3, *p* < 0.001) are factors leading potentially to the exhibition of too high blood sugar levels in hypoglycemic patients compared to non-hypoglycemic group. Taken together, these findings show that hypoglycemic patients had more often significant variations in blood sugar levels when they exhibited some of the analyzed behaviors.

## 4. Discussion

The morbidity and mortality of diabetes mellitus are increasing year by year worldwide. In 2018 there were 30.2 million (12.2%) U.S. adults affected by this disease [23]. Most of them (83%) were threatened by glucose-lowering pharmacotherapy, leading to iatrogenic hypoglycemia, a treatment-limited adverse effect [23,24]. This condition leads to increased mortality, impaired quality of life, high costs, and diminished productivity [25,26,27,28]. In our study, we analyzed and compared multidimensional data from hypoglycemic patients and found factors linked to the increased risk of hypoglycemia. The rate of exhibiting hypoglycemic episodes was 62.0% (186/300). This result includes patients with symptomatic hypoglycemia; however, due to the hypoglycemia unawareness (HU) [29], this finding is likely to be much greater than we showed in our study. The presence of HU increases the risk of severe hypoglycemia (six-fold for type 1 diabetes mellitus and 17-fold for type 2 diabetes mellitus) [30].

At first, we indicated that individuals with BMI < 23.9 kg/m^2^ (according to the World Health Organization, BMI between 18.5 kg/m^2^ and 25 kg/m^2^ is considered normal-weight, and BMI of 18.5 kg/m^2^ or lower is considered underweight) are more likely to suffer from hypoglycemia (Figure 1 and Table S3). This result is in line with independent studies of Yun et al. and Tsai et al., who evidenced that underweight patients with type 2 diabetes have hypoglycemia more often than normal-weight individuals [31,32]. With assessed in our study cut-off of BMI < 23.9 kg/m^2^, we could not indicate the number and percentage of normal-weight and underweight individuals among our study group. Nevertheless, we suggest the significant correlation between BMI and hypoglycemia frequency may partially result from coexisting conditions, such as malnutrition, which is associated with increased morbidity and mortality in diabetic patients [33].

Furthermore, among our study population, 36 individuals used internet-connected smartphones and/or computers (36/186; 19.4%, Table S2). According to a survey conducted in February 2021 in the United States, nearly half of the respondents stated that, on average, they spend daily five to six hours on their mobile devices [34]. Together with our findings, this data raises many doubts as a gradually increasing public health problem and brings an overall increase in screen-based sedentary behavior [35]. Beyond altering energy expenditure by displacing time spent on physical activities, too much screen time is associated with unhealthy eating (e.g., higher intake of fried foods, processed meat, and sugar-sweetened beverages and a lower intake of fruits, vegetables, and whole grains), increasing the risk of hypoglycemia episodes. Furthermore, spending time on mobile devices may be associated with the intake of advertised foods or beverages and may attract some individuals to begin smoking, additionally harming the life quality of hypoglycemic patients [36].

Among our study population, 57 hypoglycemic patients did not follow any recommendations regarding non-pharmacology treatment, such as physical activity and adequate diet (57/186; 30.6%, Table S2 and *p* = 0.03; OR = 2.07, CI95% [1.07–4.00], Table S3 and Figure 1). A sedentary lifestyle, enhanced by using tablets, computers, smartphones, etc., even if used to learn about diabetes mellitus, increases the risk of hypoglycemia by disrupting the maintenance of proper blood glucose levels. According to our findings, 99 hypoglycemic patients declared they would use mobile medical applications if they were free and accessible in online stores (99/186; 53.2%, *p* = 0.004; Table S2). This behavior increases the risk of hypoglycemia approximately two times. The 2018 Physical Activity Guideline released by the CDC suggests sitting less and moving more, even if we have good intentions to do so, and these behaviors do not result from laziness [37]. Moreover, according to our findings, we identified novel factors of hypoglycemia such as the lack of trust in medical content, interpretation of laboratory tests, and description of ailments available on the internet, and the statement that the best source to learn about diabetes is to gain information from friends and/or family members (Figure 1, Table S3). This behavior is the most significant in patients with type 2 diabetes, for whom the risk of hypoglycemia increases almost six times (*p* = 0.006; OR = 6.04, CI95% [1.68–21.7], Table S3b). These actions may lead to a misleading view of diabetes epidemiology, treatment, and prevention. Therefore, it is recommended to use only reliable and proven scientific sources [38].

A significant correlation was also observed among individuals with unhealthy alcohol drinking behaviors (Figure 3C, *p* = 0.002) and smokers (Figure 3D, *p* = 0.002). These findings are consistent with other studies, showing that ethanol, cigarettes, and other smoking products induce hypoglycemia [39,40]. Heavy drinking contributes to the induction of pancreatitis, disturbance of the carbohydrate and glucose metabolism, and impaired liver function, which affects the blood glucose levels and results in hypoglycemia [41]. The most profound impact of smoking is associated with insulin sensitivity. Smoking decreases subcutaneous absorption of insulin, resulting in increased dosing requirements. This behavior may also alter the pathogenesis of early steps in insulin action, such as signal transduction and glucose transport, increasing the risk of micro-and macrovascular complications in patients with diabetes mellitus [42,43,44,45,46].

Hyperlipidemia (Figure 2A, *p* = 0.007), retinopathy (Figure 2B, *p* < 0.001), and asthma (Figure 2C, *p* = 0.048) were identified as common comorbidities among hypoglycemic patients. Hyperlipidemia may be a consequence of the chronic increase in blood sugar levels. This abnormality occurs in about 10% of people with type 1 diabetes and about 60–80% with type 2 diabetes [47]. Incorrect lipid profiles increase the risk of cardiovascular diseases, heart attacks, and strokes. In diabetic patients, hyperlipidemia results from the insulin resistance of adipose tissues, leading to lipid abnormalities. It is influenced by genetic and environmental factors, such as non-compliance with the diet or low physical activity, smoking, the use of certain medications, and individual factors (age, sex, body weight) [48,49]. The other significant complication of diabetes is retinopathy. This disease can lead to complete blindness. It has been shown that there is a 60% likelihood of diabetic retinopathy in patients with type 2 diabetes, and 33% of them lose their eyesight completely [49,50]. Furthermore, although there are limited data on the risk of pulmonary diseases in patients with diabetes [51], our study revealed a significant correlation between hypoglycemia and asthma (Figure 2C, *p* = 0.048). This result is in line with Ehrlich et al.’s study, which conducted a retrospective, longitudinal cohort study including more than one million members in the US to evaluate and compare the incidence of pulmonary diseases in patients with and without a diagnosis of diabetes. They found that individuals with diabetes are at increased risk of several pulmonary conditions, including asthma, chronic obstructive pulmonary disease (COPD), fibrosis, and pneumonia [52]. This observation may be a consequence of declining lung function in patients with diabetes [53].

Our study also revealed hypoglycemic patients who had the most significant fluctuations in blood glucose levels. At first, we observed that following a recommended diet, especially maintaining adequate time between meals, is crucial to controlling glucose levels. The consumption of the wrong type of meal, skipping a meal, or overeating increase fluctuations in blood glucose levels (Figure 6). This phenomenon can be significantly worsened in people with low physical activity. It is also essential to take the right insulin or other diabetes medication doses (*p* < 0.001, Figure 6). The incorrect dose may result in either hypo- or hyperglycemia. The correct dosage of insulin depends on many factors, such as the time of day and insulin resistance. Furthermore, in determining the right dosage, the strength of insulin should be considered when injecting the drug [54,55]. Furthermore, we indicated that long stress exposure increases the risk of hypoglycemia (*p* < 0.001, Figure 6). Stress leads to the release of glucose by adrenal glands, leading to increased glucose levels in the bloodstream. Repeated episodes of stress can cause severe changes in blood sugar levels, making it harder for patients with diabetics to manage their condition and increasing the risk of hypoglycemia [56,57].

It is necessary to educate and inform patients and healthcare providers about the advantages and disadvantages of glucose-lowering therapy. According to our study, 41 hypoglycemic respondents admitted their knowledge about diabetes is limited (41/186; 22.0%, *p* = 0.02, Table S2). There is clear evidence that diabetes education improves patient outcomes [29,30]. Diabetes self-management training aims to improve knowledge about biomedical, behavioral, and psychosocial processes and outcomes (e.g., risks of hypoglycemia, medication use, self-monitoring of blood glucose, dietary management, psychical activity, health beliefs, self-management skills, coping skills, distress related to diabetes, treatment satisfaction, and diabetes-specific quality of life) [58,59,60]. To date, several diabetes self-management education programs are available in different countries, including the Dose Adjustment for Normal Eating (DAFNE) program in the UK, Ireland, Australia, New Zealand, Kuwait, and Singapore [61], X-PERT and DESPOMD programs in the UK [62], SIDEP (the structured intensive diabetes education program) in South Korea and the New Zealand [63], ROMEO program in Italy [64], or Uppsala study in Sweden [65]. There are also several established programs, the results of which are reported in scientific databases (such as PubMed, Google Scholar) in Austria [66], Germany [67], Switzerland [68], and France [69].

Considering Polish healthcare systems, this country provides medical laboratory diagnostics services for detecting, monitoring, and treating diabetes, financed from public funds as part of primary health care. For instance, to improve the quality of care for diabetic patients, the National Health Fund established a contractual program: “Comprehensive specialist outpatient care for diabetic patients—KAOS [70]. Other programs and preventive services available for the Polish population are the “STOP DIABETES” program co-financed by the European Social Fund [71]; the Program for the early detection and prevention of diabetes among the inhabitants of the Mazowieckie Voivodeship for the years 2017–2019 [72]; or the international “Diabetes in Europe—Prevention through Physical Activity, Lifestyle Change and Nutritional Intervention—DE-PLAN” program [73].

Nevertheless, although years of approaches to implement numerous educational programs in Poland aiming at preventive screening services in risk groups, diabetes mellitus is still a significant public health problem. The increasing incidences of this disease result from an unhealthy lifestyle and still too low awareness about the severity of the disease. Diabetes, as a civilization disease, requires a systemic approach by creating new international, national, and local programs for combating this disease [73]. These structured education programs should be tailored to the individual, evidence-based preferences and learning styles with specific aims and objectives. Therefore, it is beneficial to find groups of diabetic patients who are in particular need of care in terms of DM. We believe that our study is a first step in implementing novel preventive programs for severe hypoglycemic patients in Poland.

There are several limitations to our study. First of all, the cross-sectional nature of this study impeded any conclusion about causal relations, making it challenging to draw firm assumptions about the direction of exposure-outcome associations. Secondly, the limited sample size (*n* = 300) yielded broad confidence intervals with the risk of overlooking associated characteristics. Last but not least, the possibility of a recall bias cannot be ruled out in self-reports of self-management, making the findings reliant upon the accuracy of the patient’s self-evaluation.

To summarize, our study showed that many clinical aspects and risk factors correlate significantly with the occurrence of hypoglycemia. Understanding the interactions between diabetes and sociodemographic, environmental, or other disease-related risk factors may provide new strategies to optimize glycemic control and reduce the occurrence of hypoglycemia.

## Data Availability

The authors confirm that the data supporting the findings of this study are available within the article.

## Supplementary Materials

The following supporting information can be downloaded at: https://www.mdpi.com/article/10.3390/jcm11030626/s1, Table S1: General and clinical characteristics of diabetic patients based on the characteristics of patients with frequent hypoglycemia and the odds ratios of hypoglycemia and their 95% confidence intervals; (a) general and clinical characteristics of patients with type 1 diabetes; (b) general and clinical characteristics of patients with type 2 diabetes; Table S2: Number (*n*) and percentage (%) of respondents in groups that differed in the frequency of symptoms of hypoglycemia and in the responses to the survey questions; (a) number (*n*) and percentage (%) of patients with type 1 diabetes in groups differing in the frequency of symptoms of hypoglycemia and the answers to the questionnaire; (b) number (*n*) and percentage (%) of patients with type 2 diabetes in groups differing in the frequency of symptoms of hypoglycemia and the answers to the questionnaire; Table S3: Results of multivariate logistic regression analysis in the entire group of hypoglycemic respondents (*n* = 186); (a) the results from the multivariate logistic regression analysis of patients with type 1 diabetes in the group of hypoglycemic respondents (*n* = 116), (b) The results of the multivariate logistic regression analysis of patients with type 2 diabetes in the group of hypoglycemic respondents (*n* = 184).
