# Supplementary material for: The Impact of Hypoglycemia on Patients with Diabetes Mellitus: A Cross-Sectional Analysis
Source: J Clin Med. 2022 Jan 26;11(3):626. doi: 10.3390/jcm11030626 (PMC8836583; doi:10.3390/jcm11030626)
Supplement: Supplementary file 1 [file jcm-11-00626-s001.zip › jcm-1522127-supplementary.pdf]

Table S1. General and clinical characteristics of diabetic patients based on the characteristics of patients with frequent hypoglycemia and the odds ratios of hypoglycemia and their 95% confidence intervals.

Predictors of hypoglycemia	Common symptoms				*p*	OR (95% CI)
	Yes *n* = 186		No *n* = 114			
	n	%	*n*	%		
Male	102	54.8	54	47.4	0.209	1.35 (0.85–2.15)
Age < 55 years	111	59.7	62	54.4	0.368	1.24 (0.78–1.99)
Live in a city with less than 500,000 inhabitants	168	90.3	99	86.8	0.350	1.41 (6.68–2.93)
At least post-secondary education	157	84.4	94	82.5	0.657	1.15 (0.62–2.15)
Net income < 2000 PLN	96	57.5	53	52.5	0.424	1.22 (0.75–2.01)
BMI < 23.9 kg/m^2	89	47.8	38	33.3	**0.014**	**1.84 (1.13–2.98)**
Type 1 diabetes	124	66.7	63	55.3	**0.048**	**1.62 (1.00–2.61)**
Time since diagnosis ≥ 8 years	77	41.4	38	33.3	0.163	1.41 (0.87–2.30)
Hypertension	82	44.1	49	43.0	0.852	1.05 (0.65–1.67)
Overweight/obesity	49	26.3	33	29.0	0.623	0.88 (0.52–1.48)
Osteoporosis	20	10.8	9	7.9	0.541	1.41 (0.62–3.20)
Asthma	45	24.2	16	14.0	**0.048**	**1.95 (1.05–3.66)**
Another chronic disease	29	15.6	18	15.8	0.963	0.99 (0.52–1.87)

Table S1a. General and clinical characteristics of patients with type 1 diabetes based on the characteristics of patients with frequent hypoglycemia and the odds ratios of hypoglycemia and their 95% confidence intervals

Hypoglycemia predictors in the group of patients with type 1 diabetes	Common symptoms				*p*	OR (95% CI)
	Yes *n* = 79		No *n* = 37			
	n	%	*n*	%		
Male	45	57.0	57	53.3	0.656	1.16 (0.65–2.08)
Age < 55 years	48	60.8	20	54.1	0.494	1.32 (0.60–2.90)
Live in a city with less than 500,000 inhab.	75	94.9	93	86.9	0.081	2.82 (0.89–8.93)
At least post-secondary education	66	83.5	29	78.4	0.606	1.40 (0.52–3.74)
Net income < 2000 PLN	46	58.2	18	48.6	0.423	1.47 (0.67–3.22)
BMI < 23.9 kg/m^2	33	41.8	11	29.7	0.227	1.70 (0.74–3.91)
Time since diagnosis ≥ 8 years	40	50.6	18	48.6	1.000	1.08 (0.50–2.36)
Hypertension	36	45.6	18	48.6	0.842	0.88 (0.40–1.93)
Overweight/obesity	26	32.9	9	24.3	0.392	1.53 (0.63–3.70)
Osteoporosis	3	3.8	4	10.8	0.207	0.33 (0.07–1.54)
Asthma	16	20.3	4	10.8	0.293	2.10 (0.65–6.78)
Another chronic disease	14	17.7	7	18.9	0.876	0.92 (0.34–2.52)

Table S1b. General and clinical characteristics of patients with type 2 diabetes based on the characteristics of patients with frequent hypoglycemia and the odds ratios of hypoglycemia and their 95% confidence intervals

Hypoglycemia predictors in the group of patients with type 2 diabetes	Common symptoms				*p*	OR (95% CI)
	Yes *n* = 107		No *n* = 77			
	n	%	*n*	%		
Male	57	53.3	35	45.5	0.370	1.37 (0.76–2.46)
Age < 55 years	63	58.9	42	54.5	0.651	1.19 (0.66–2.16)
Live in a city with less than 500,000 inhab.	93	86.9	69	89.6	0.650	0.77 (0.31–1.94)
At least post-secondary education	91	85.0	65	84.4	1.000	1.05 (0.47–2.37)
Net income < 2000 PLN	50	46.7	35	45.5	0.882	1.05 (0.58–1.89)
BMI < 23.9 kg/m^2	56	52.3	27	35.1	**0.024**	**2.03 (1.11–3.71)**
Time since diagnosis ≥ 8 years	37	34.6	20	26.0	0.259	1.51 (0.79–2.88)

Hypertension	46	43.0	31	40.3	0.763	1.12 (0.62–2.03)
Overweight/obesity	23	21.5	24	31.2	0.171	0.60 (0.31–1.18)
Osteoporosis	17	15.9	5	6.5	0.066	2.72 (0.96–7.73)
Asthma	29	27.1	12	15.6	0.074	2.01 (0.95–4.26)
Another chronic disease	15	14.0	11	14.3	1.000	0.98 (0.42–2.27)

Table S2. Number (*n*) and percentage (%) of respondents in groups that differed in the frequency of symptoms of hypoglycemia and in the responses to the survey questions.

Answers to survey questions	Common symptoms				*p*	OR (95% CI)
	Yes $n = 186$		No $n = 114$			
	n	%	*n*	%		
1. How satisfied are you with the medical care you receive for your illness? (scale from 0—definitely dissatisfied to 10—definitely satisfied): "<5 pts."	52	28.0	20	17.5	**0.040**	**1.82 (1.02–3.25)**
2. How often do you use your internet-connected smartphone and/or computer? "Not every day."	36	19.4	12	10.5	**0.043**	**2.04 (1.01–4.11)**
3. If there was a free mobile application that could be installed on your smartphone and carried out intelligent monitoring of the state of your disease and recommended appropriate actions based on scientific grounds, would you use it ?: "<5 pts."	26	14.0	6	5.3	**0.029**	**2.92 (1.16–7.34)**
4. Which sentence describes your situation best: "I visit different doctors in one health center/clinic, where the records of my disease are kept","I visit different doctors who have access to the electronic version of my disease records" and "I go to different doctors with the documentation I keep (in paper version) about my disease".	86	46.2	34	29.8	**0.005**	**2.02 (1.23–3.32)**
5. If there was an application that allows you to store the history of laboratory test results in an electronic form (organized, secured, and accessible via the Internet), how likely is it that you would use it?:"> 5 pts."	72	38.7	36	31.6	0.212	1.37 (0.84–2.24)
6. Currently uses the available mobile applications in the topic of health monitoring	35	18.8	17	14.9	0.386	1.32 (0.70–2.49)
7. Which would positively influence your decision to start using the free health monitoring app (on a scale of 0–10)?						
7 a) The application would be free and available in popular online stores: "< 9"	99	53.2	41	36.0	**0.004**	**2.03 (1.25–3.27)**
7 b) The application would be recommended by the National Health Fund or the Social Insurance Institution: "< 10"	126	67.7	70	61.4	0.263	1.32 (0.81–2.15)

7 c) The application would be recommended as part of private medical care packages: "< 10"	144	77.4	84	73.7	0.462	1.22 (0.71–2.10)
7 d) My doctor recommends using the app: "< 10"	135	72.6	75	65.8	0.213	1.38 (0.83–2.28)
7 e) The application would give discounts to insurance companies when buying a policy, e.g., for life or health insurance: "< 10"	135	72.6	72	63.2	0.087	1.54 (0.94–2.54)
8. The app would provide me with advanced interpretations of lab results and guidelines for my actions to maintain good health: "< 10"	128	68.8	63	55.3	**0.018**	**1.79 (1.10–2.89)**
9 a) Self-assessment of knowledge about diabetes < 6 pts.	41	22.0	13	11.4	**0.020**	**2.20 (1.12–4.31)**
9 b) The level of trust in medical content, interpretation of laboratory tests and description of ailments available on the Internet < 5 pts.	31	16.7	9	7.9	**0.046**	**2.33 (1.07–5.10)**
10. What sources do you learn about the disease and how to proceed:						
10 a) From diabetic training: "Rather yes" or "Definitely yes"	80	43.0	39	34.2	0.130	1.45 (0.89–2.35)
10 b) From the attending physician: "Definitely no", "Probably not", "Moderately" or "Rather yes"	86	46.2	41	36.0	0.081	1.53 (0.95–2.47)
10 c) From books and articles about diabetes: "Definitely not", "Rather not" or "Moderately"	77	41.4	35	30.7	0.063	1.59 (0.97–2.61)
10 d) From the websites: "Definitely not", "Rather not" or "Moderately"	86	46.2	41	36.0	0.081	1.53 (0.95–2.47)
10 e) From Internet forums, Faceboo,k et.c: "Rather yes" or "Definitely yes"	75	40.3	41	36.0	0.452	1.20 (0.74–1.95)
10 f) From seminars and conferences: "Moderately", "Rather yes" or "Definitely yes"	102	54.8	48	42.1	**0.032**	**1.67 (1.04–2.67)**
10 g) From other people—friends, family: "Definitely yes"	43	23.1	9	7.9	**0.001**	**3.51 (1.64–7.51)**
11. If the application for monitoring the health condition contained an extensive and precise description of the disease and instructions on how to proceed, would you be prompted to install it?: "Definitely not", "Not really" or "I don't know / hard to say"	57	30.6	25	21.9	0.100	1.57 (0.91–2.71)
12. Are your glycosylated hemoglobin results normal?: "Yes"	153	82.3	88	86.8	0.293	0.70 (0.36–1.36)
13. Have you had a follow-up appointment with a diabetologist in the last year? "I have never been to a diabetologist"	7	3.8	2	1.8	0.491	2.19 (0.45–10.7)
14. Do you follow any recommendations regarding non-pharmacological treatment,	57	30.6	19	16.7	**0.007**	**2.21 (1.23–3.96**

in particular regarding physical activity and a proper diet? "No"						
15. How do you rate your health? "Not very good", "Bad" or "Very bad"	84	45.2	36	31.6	**0.020**	**1.78 (1.09–2.91)**
16. How often have you had severe low blood sugar event in the last year, including passing out or needing help?: "At least once"	163	87.6	0	0.0	**<0.001**	**-**
17. In the last month, how many days have you had high blood sugar with symptoms such as thirst, dry mouth and skin, increased urine sugar, decreased appetite, nausea or tiredness?: "4 days or more"	69	37.1	12	10.5	**<0.001**	**5.01 (2.57–9.78)**

Table S2a. Number (*n*) and percentage (%) of patients with type 1 diabetes in groups differing in the frequency of symptoms of hypoglycaemia and the answers to the questionnaire

Answers to survey questions	Common symptoms				*p*	OR (95% CI)
	Yes *n* = 79		No *n* = 37			
	n	%	*n*	%		
1. How satisfied are you with the medical care you receive for your illness? (scale from 0—definitely dissatisfied to 10—definitely satisfied): "<5 pts."	20	25.3	6	16.2	0.343	1.75 (0.64–4.81)
2. How often do you use your internet-connected smartphone and/or computer?: "Not every day"	33	41.8	10	27.0	0.151	1.94 (0.83–4.54)
3. If there was a free mobile application that could be installed on your smartphone and carried out intelligent monitoring of the state of your disease and recommended appropriate actions based on scientific grounds, would you use it ?: "<5 pts."	9	11.4	3	8.1	0.749	1.46 (0.37–5.73)
4. I visit different doctors in one health center/clinic, where the records of my disease are kept" or "I visit different doctors who have access to the electronic version of my disease records" or "I go to different doctors with the documentation I keep (in paper version) about my disease"	73	92.4	34	91.9	1.000	1.07 (0.25–4.55)
5. If there was an application that allows you to store the history of laboratory test results in an electronic form (organized, secured and accessible via the Internet), how likely is it that you would use it ?:"> 5 pts."	59	89.4	29	87.9	1.000	1.16 (0.31–4.29)
6. Currently uses the available mobile applications in the topic of health monitoring	13	16.5	5	13.5	0.788	1.26 (0.41–3.84)

7. Which would positively influence your decision to start using the free health monitoring app (on a scale of 0–10)?						
7 a) The application would be free and available in popular online stores: "< 9"	36	45.6	12	32.4	0.226	1.74 (0.77–3.95)
7 b) The application would be recommended by the National Health Fund or the Social Insurance Institution: "< 10"	51	64.6	21	56.8	0.538	1.39 (0.63–3.08)
7 c) The application would be recommended as part of private medical care packages: "< 10"	57	72.2	26	70.3	0.829	1.10 (0.46–2.59)
7 d) My doctor recommends using the app: "< 10"	54	68.4	24	64.9	0.832	1.17 (0.51–2.67)
7 e) The application would give discounts to insurance companies when buying a policy, e.g., for life or health insurance: "< 10"	50	63.3	20	54.1	0.416	1.47 (0.66–3.24)
8. The app would provide me with advanced interpretations of lab results and guidelines for my actions to maintain good health: "< 10"	46	58.2	18	48.6	0.423	1.47 (0.67–3.22)
9 a) Self-assessment of knowledge about diabetes < 6 pts.	14	17.7	4	10.8	0.418	1.78 (0.54–5.83)
9 b) The level of trust in medical content, interpretation of laboratory tests and d,escription of ailments available on the Internet.	13	16.5	6	16.2	1.000	1.02 (0.35–2.93)
10. What sources do you learn about the disease and how to proceed:						
10 a) From diabetic training: "Rather yes" or "Definitely yes"	32	40.5	11	29.7	0.306	1.61 (0.70–3.71)
10 b) From the attending physician: "Definitely no", "Probably not", "Moderately" or "Rather yes"	29	36.7	16	43.2	0.543	0.76 (0.34–1.69)
10 c) From books and articles about diabetes: "Definitely not", ","ther not" or "Moderately"	31	39.2	13	35.1	0.838	1.19 (0.53–2.69)
10 d) From the websites: "Definitely not", "Rather not" or "Moderately"	37	46.8	8	21.6	**0.014**	**3.19 (1.30–7.85)**
10 e) From Internet forums, Facebook etc: "Rather yes" or "Definitely yes"	29	36.7	16	43.2	0.543	0.76 (0.34–1.69)
10 f) From seminars and conferences: "Moderately", "Rather yes" or "Definitely yes"	40	50.6	16	43.2	0.551	1.35 (0.61–2.95)
10 g) From other people—friends, family: "Definitely yes"	15	19.0	4	10.8	0.420	1.93 (0.59–6.29)
11. If the application for monitoring the health condition contained an extensive and precise description of the disease and instructions on how to proceed, would you	19	24.1	9	24.3	1.000	0.99 (0.40–2.45)

be prompted to install it?: "Definitely not", "Not really" or "I don't know / hard to say"						
12. Are your glycosylated hemoglobin results normal ?: "Yes"	64	81.0	33	89.2	0.420	0.52 (0.16–1.68)
13. Have you had a follow-up appointment with a diabetologist in the last year?: "I have never been to a diabetologist"	3	3.8	0	0.0	0.550	-
14. Do you follow any recommendations regarding non-pharmacological treatment, in particular regarding physical activity and a proper diet? "No"	26	32.9	6	16.2	0.076	2.53 (0.94–6.84)
15. How do you rate your health? "Not very good", "Bad" or "Very bad"	37	46.8	13	35.1	0.315	1.63 (0.73–3.64)
16. In the last year, how many times have you had severe low blood sugar events, including passing out or needing help?: "At least once"	70	88.6	0	0.0	**<0.001**	-
17. In the last month, how many days have you had high blood sugar with symptoms such as thirst, dry mouth and skin, increased urine sugar, decreased appetite, nausea or tiredness? "4 days or more"	36	45.6	6	16.2	**0.002**	**4.33 (1.62–11.5)**

Table S2b. Number (*n*) and percentage (%) of patients with type 2 diabetes in groups differing in the frequency of symptoms of hypoglycaemia and the answers to the questionnaire

Answers to survey questions	Common symptoms				*p*	OR (95% CI)
	Yes *n* = 107		No *n* = 77			
	n	%	*n*	%		
1. How satisfied are you with the medical care you receive for your illness? (scale from 0—definitely dissatisfied to 10—definitely satisfied): "<5 pts."	32	29.9	14	18.2	0.085	1.92 (0.94–3.91)
2. How often do you use your internet-connected smartphone and/or computer? "Not every day"	52	48.6	18	23.4	**0.001**	**3.10 (1.62–5.94)**
3. If there was a free mobile application that could be installed on your smartphone and carried out intelligent monitoring of the state of your disease and recommended appropriate actions based on scientific grounds, would you use it ?: "<5 pts."	17	15.9	3	3.9	**0.015**	**4.66 (1.31–16.5)**
4. I visit different doctors in one health center / clinic / clinic, where the records of my disease are kept" or "I visit different doctors who have access to the electronic version of my disease records" or "I go to different doctors with the documentation I keep (in paper version) about my disease"	99	92.5	69	89.6	0.598	1.43 (0.51–4.01)

5. If there was an application that allows you to store the history of laboratory test results in an electronic form (organized, secured and accessible via the Internet), how likely is it that you would use it ?:"> 5 pts."	78	95.1	52	80.0	**0.008**	**4.88 (1.51–15.8)**
6. Currently uses the available mobile applications in the topic of health monitoring	22	20.6	12	15.6	0.445	1.40 (0.65–3.04)
7. Which would positively influence your decision to start using the free health monitoring app (on a scale of 0–10)?						
7 a) The application would be free and available in popular online stores: "< 9"	63	58.9	29	37.7	**0.007**	**2.37 (1.30–4.32)**
7 b) The application would be recommended by the National Health Fund or the Social Insurance Institution: "< 10"	75	70.1	49	63.6	0.426	1.34 (0.72–2.49)
7 c) The application would be recommended as part of private medical care packages: "< 10"	87	81.3	58	75.3	0.363	1.43 (0.70–2.90)
7 d) My doctor recommends using the app: "< 10"	81	75.7	51	66.2	0.185	1.59 (0.83–3.03)
7 e) The application would give discounts to insurance companies when buying a policy, e.g. for life or health insurance: "< 10"	85	79.4	52	67.5	0.086	1.86 (0.95–3.63)
8. The app would provide me with advanced interpretations of lab results and guidelines for my actions to maintain good health: "< 10"	82	76.6	45	58.4	**0.010**	**2.33 (1.23–4.41)**
9 a) Self-assessment of knowledge about diabetes < 6 pts.	27	25.2	9	11.7	**0.024**	**2.55 (1.12–5.79)**
9 b) The level of trust in medical content, interpretation of laboratory tests and description of ailments available on the Internet < 5 pts.	18	16.8	3	3.9	**0.008**	**4.99 (1.41–17.6)**
10. What sources do you learn about the disease and how to proceed:						
10 a) From diabetic training: "Rather yes" or "Definitely yes"	48	44.9	28	36.4	0.289	1.42 (0.78–2.60)
10 b) From the attending physician: "Definitely no", "Probably not", "Moderately" or "Rather yes"	57	53.3	25	32.5	**0.007**	**2.37 (1.29–4.36)**
10 c) From books and articles about diabetes: "Definitely not", "Rather not" or "Moderately"	46	43.0	22	28.6	**0.046**	**1.89 (1.01–3.52)**
10 d) From the websites: "Definitely not", "Rather not" or "Moderately"	49	45.8	33	42.9	0.764	1.13 (0.62–2.03)
10 e) From Internet forums, Facebook etc: "Rather yes" or "Definitely yes"	46	43.0	25	32.5	0.169	1.57 (0.85–2.89)

10 f) From seminars and conferences: "Moderately", "Rather yes" or "Definitely yes"	62	57.9	32	41.6	**0.036**	**1.94 (1.07–3.51)**
10 g) From other people—friends, family: "Definitely yes"	28	26.2	5	6.5	**0.001**	**5.10 (1.87–13.9)**
11. If the application for monitoring the health condition contained an extensive and precise description of the disease and instructions on how to proceed, would you be prompted to install it?: "Definitely not", "Not really" or "I don't know / hard to say"	38	35.5	16	20.8	**0.034**	**2.10 (1.07–4.14)**
12. Are your glycosylated hemoglobin results normal?: "Yes"	89	83.2	66	85.7	0.687	0.82 (0.36–1.86)
13. Have you had a follow-up appointment with a diabetologist in the last year?: "I have never been to a diabetologist"	4	3.7	2	2.6	1.000	1.46 (0.26–8.16)
14. Do you follow any recommendations regarding non-pharmacological treatment, in particular regarding physical activity and a proper diet? "No"	31	29.0	13	16.9	0.079	2.01 (0.97–4.16)
15. How do you rate your health? "Not very good", "Bad" or "Very bad"	47	43.9	23	29.9	0.065	1.84 (0.99–3.42)
16. In the last year, how many times have you had severe low blood sugar events, including passing out or needing help?: "At least once"	93	86.9	0	0.0	**<0.001**	-
17. In the last month, how many days have you had high blood sugar with symptoms such as thirst, dry mouth and skin, increased urine sugar, decreased appetite, nausea or tiredness? "4 days or more"	33	30.8	6	7.8	**<0.001**	**5.28 (2.08–13.4)**

Table S3. Results of multivariate logistic regression analysis in the entire group of hypoglycemic respondents (*n*=186) including data from table S1 – sociodemographic factors, other prevailing chronic diseases and table S2—responses to the surveyed questions regarding the willingness to use mobile medical applications, statements about the trust in medical services in Poland and the level of knowledge about diabetes, and. As all the shown independent variables are binary (1 = yes, 0 + no), the magnitude of the influence of the variables on the likelihood to have hypoglycemia is evidenced by their value.

Predictors of hypoglycemia	Multivariate		
	b	*p*	OR (95% CI)
• BMI < 23.9	0.850	0.003	2.34 (1.34–4.08)
• Visits of different doctors	0.722	0.011	2.06 (1.18–3.60)
• Access to free mobile medical applications in online stores	0.643	0.022	1.90 (1.10–3.30)
• Lack of trust in medical content, interpretation of laboratory tests, and description of ailments available on the Internet	0.919	0.037	2.51 (1.06–5.94)

• Statement that the best source to learn about diabetes is from friends and/or family members	1.353	0.001	3.87 (1.68–8.88)
• Lack of physical activity and/or proper diet	0.729	0.030	2.07 (1.07–4.00)
• Hypoglycemic symptoms occurring continuously four days and/or more in the last month	1.670	<0.001	5.31 (2.60–10.9)

* All logistic regression coefficients ("b") in this model are positive, indicating that all these factors increase the likelihood of a hypoglycemia.

Table S3a. The results of the multivariate logistic regression analysis of patients with type 1 diabetes in the group of hypoglycemic respondents (*n* = 116), taking into account the data from Table S1a—sociodemographic factors, other dominant chronic diseases and Table S2a—answers to the surveyed questions about the willingness to use mobile medical applications, statements about trust in medical services in Poland and the level of knowledge about diabetes. Since all of the independent variables presented are binary (1 = yes, 0 + no), the magnitude of the influence of these variables on the probability of hypoglycaemia is indicated by their value.

Predictors of hypoglycemia	Multivariate		
	b	*p*	OR (95% CI)
• Statement that the best source to learn about diabetes is from websites	1.024	0.030	2.79 (1.10–7.04)
• Symptoms such as thirst, dry mouth and skin, increased urine sugar, decreased appetite, nausea or tiredness occurring continuously four days and/or more in the last month	1.352	0.008	3.87 (1.42–10.5)

Table S3b. The results of the multivariate logistic regression analysis of patients with type 2 diabetes in the group of hypoglycemic respondents (*n* = 184), taking into account the data from Table S1a—sociodemographic factors, other dominant chronic diseases and Table S2a—answers to the surveyed questions about the willingness to use mobile medical applications, statements about trust in medical services in Poland and the level of knowledge about diabetes. Since all of the independent variables presented are binary (1 = yes, 0 + no), the magnitude of the influence of these variables on the probability of hypoglycaemia is indicated by their value.

Predictors of hypoglycemia	Multivariate		
	b	*p*	OR (95% CI)
• BMI < 23.9 kg/m^2	**−1.376**	**0.003**	**0.25 (0.10–0.63)**
• The use of internet-connected smartphone and/or computer less often than every day	**1.179**	**0.012**	**3.25 (1.30–8.15)**
• The use of an application that allows to store the history of laboratory test results in an electronic form (organized, secured and accessible via the Internet)	**1.525**	**0.036**	**4.60 (1.10–19.2)**
• The agreement to use health monitoring apps if they are free and available in popular online stores	**1.178**	**0.011**	**3.25 (1.31–8.06)**

• The use of application that provide with advanced interpretations of lab results and guidelines to maintain good health	−0.137	0.801	0.87 (0.30–2.53)
• The lack of knowledge about diabetes	0.394	0.547	1.48 (0.41–5.35)
• The low level of trust in medical content, interpretation of laboratory tests and description of ailments available on the Internet	1.419	0.146	4.13 (0.61–28.0)
• Statement that the best source to learn about diabetes is from attending physician	**1.140**	**0.013**	**3.13 (1.27–7.68)**
• Statement that the best source to learn about diabetes is from attending physician	0.196	0.699	1.22 (0.45–3.30)
• Statement that the best source to learn about diabetes is from seminars and conferences	0.275	0.538	1.32 (0.55–3.16)
• Statement that the best source to learn about diabetes is from friends and/or family	**1.799**	**0.006**	**6.04 (1.68–21.7)**
• The agreement to install the application for monitoring the health condition only if it contains an extensive and precise description of the disease and instructions on how to proceed	0.484	0.444	1.62 (0.47–5.61)
• Symptoms such as thirst, dry mouth and skin, increased urine sugar, decreased appetite, nausea or tiredness occurring continuously four days and/or more in the last month	**2.142**	**0.001**	**8.52 (2.30–31.5)**
